# Single-cell transcriptome atlas of testes from mice with high-fat diets

**DOI:** 10.1038/s41597-024-03435-5

**Published:** 2024-06-04

**Authors:** Wenbin Cao, Yulin Zhang, Jia Qi, Yanru Zhang, Ruike Ding, Bin Meng, Juan Zhao, Shiwei Luo, Chong Shen, Chenjin Duan, Hongyu Qin, Yun Ye, Enqi Liu, Pengxiang Qu

**Affiliations:** 1 Laboratory Animal Center, School of Basic Science, Xi’an Jiaotong University, 76 Yanta West Road, Xi’an, Shaanxi 710061 China; 2 Key Laboratory of Environment and Genes Related to Diseases, Ministry of Education of China, Xi’an, 710049 China; 3 Center for Reproductive Medicine, Xi’an Angel Women’s & Children’s Hospital, Xi’an, 710000 China; 4 Department of Hematology, The First Affiliated Hospital of Xi’an Jiaotong University, Xi’an, Shaanxi 710061 People’s Republic of China; 5 Central Laboratory, The First Affiliated Hospital of Xi’an Medical University, Xi’an, 710000 China

**Keywords:** Nutrition disorders, Reproductive biology, Endocrine reproductive disorders, Disease model, Infertility

## Abstract

Obesity is accompanied by multiple known health risks and increased morbidity, and obese men display reduced reproductive health. However, the impact of obesity on the testes at the molecular levels remain inadequately explored. This is partially attributed to the lack of monitoring tools for tracking alterations within cell clusters in testes associated with obesity. Here, we utilized single-cell RNA sequencing to analyze over 70,000 cells from testes of obese and lean mice, and to study changes related to obesity in non-spermatogenic cells and spermatogenesis. The Testicular Library encompasses all non-spermatogenic cells and spermatogenic cells spanning from spermatogonia to spermatozoa, which will significantly aid in characterizing alterations in cellular niches and the testicular microenvironment during high-fat diet (HFD)-induced obesity. This comprehensive dataset is indispensable for studying how HFD disrupts cell-cell communication networks within the testis and impacts alterations in the testicular microenvironment that regulate spermatogenesis. Being the inaugural dataset of single-cell RNA-seq in the testes of diet-induced obese (DIO) mice, this holds the potential to offer innovative insights and directions in the realm of single-cell transcriptomics concerning male reproductive injury associated with HFD.

## Background & Summary

More than 650 million adults worldwide suffer from obesity, and more than 38 million children under the age of five are considered overweight or obese^1,2^. Obesity is also a common problem among males of reproductive age, because it can cause or exacerbate male-factor infertility through endocrine abnormalities, obesity-related problems such as loss of sperm motility, or a direct impact on the fidelity of chromosome segregation at meiotic divisions. Based on population studies, the prevalence of abnormal sperm parameters in overweight and obese males has increased^3^, with a higher risk of infertility^4^. A negative impact of male obesity on assisted reproduction has been confirmed, and a high body mass index is associated with a significant decrease in the rates of clinical pregnancies and live births during IVF/ICSI cycles^5^.

The impact of obesity on fertility has recently received increased attention, and many important findings have been reported. In spite of that, the understanding of the impact of obesity on testicular cells is still limited due to the heterogeneity of genetic or epigenetic information in these cells. The single-cell RNA sequencing (scRNA-seq) allows researchers to visualize transient gene expression, resolve characteristics of single cells, describe intercellular communication, and reveal intercellular heterogeneity^6^. Recent studies have used scRNA-seq to identify the major spermatogenic and somatic cell types in humans, monkeys, and mice, allowing the establishment of spermatogonial stem cell banking and revealing differentiation markers, potential meiotic regulatory factors, and spermatogenic cell-somatic cell communication mechanisms^7^. However, a single-cell atlas of testes from obese subjects or those on a HFD is still lacking, which limits our understanding of the impact of obesity on reproductive disorders.

A catalog of the different cell types and functions within highly organized testis in obesity would be useful, and here we used scRNA-seq to unravel the heterogeneity and complexity of RNA transcripts in an obese mouse model. The data revealed molecular events and regulatory mechanisms of gene expression at the single-cell level that occur in the testes of obese mice. The dataset captured discernible changes in the cellular niche of non-spermatogenic cells affected by HFD. Additionally, it encompassed the complete stages of spermatogenesis, ensuring the high quality and reliability of the data collected. This dataset offers comprehensive insights into the effects of HFD-induced obesity on germ cell characterization. It facilitates the identification of relevant biomarkers, the analysis of cyclic expression profiles, and the elucidation of key genes critical for spermatogenesis. Moreover, this research significantly contributes to the discovery of novel subpopulations of testicular somatic cells and enhances our understanding of the paracrine regulatory networks within the testicular microenvironment. This research will yield novel, comprehensive insights, offering directional guidance for elucidating the mechanisms underlying spermatogenesis impairment and the alterations in cellular niches induced by DIO.

## Methods

### Animals

ICR male mice purchased from the Laboratory Animal Centre of Xi’an Jiaotong University Health Science Centre (Xi’an, China), were kept on a 12 h light/12 h dark cycle, 23 ± 1 °C, and 55 ± 10% humidity. Mice were randomly assigned to two groups at the age of 6 weeks, with different diets for 12 weeks: a chow diet group (CD, D12450B, Research Diets, New Brunswick, NJ, USA) and a HFD (D12492, Research Diets). After 12 weeks, mice were euthanized, and testes were removed. All experimental procedures were approved (approval number: XJTUAE2023-2095) by the Laboratory Animal Administration Committee of Xi’an Jiaotong University, and performed in accordance with the Guidelines of the Biomedical Ethics Committee of Health Science Center of Xi’an Jiaotong University.

### Testis cell isolation and preparation

Tissue specimens, consisting of three biological replicates from the CD and HFD groups, were processed through a series of steps including dissociation, filtration, and erythrocyte lysis. Testes were sectioned and digested using sCelLive™ Tissue Dissociation Solution in the Singleron PythoN™ Tissue Dissociation System at 37 °C for 15 minutes. The resulting cell suspension was then strained through a 40-micron filter. For red blood cell removal, it was mixed with GEXSCOPE® RCLB in a 1:2 ratio and incubated at room temperature for 5–8 minutes. This procedure was pivotal in obtaining high-quality single-cell suspensions. The quantification of cellular concentration and viability was executed using Trypan blue staining, which was employed to verify the quality of the samples. The predefined quality control criteria for our study required a minimum cell viability threshold of 85%, and the total cell count needed to surpass 20,000 to ensure adequate sample size for reliable analysis.

### Library construction

Utilizing the SCOPE-chip microfluidic technology in synergy with the Singleron Matrix® Single Cell Processing System to capture individual cells. Cells were lysed in microtiter wells and mRNA was released, which was captured and immobilized on Barcoding Beads. The mRNA was reverse transcribed to obtain cDNA, which was amplified by PCR. The amplified cDNA is then fragmented and ligated with sequencing adapters. Subsequently, the construction of the library was conducted, adhering to the protocols outlined by the GEXSCOPE® Single Cell RNA Library Kits (Singleron)^8^. For effective quality control of expression libraries, the following criteria must be met: The total yield as measured by the Qubit assay should exceed 100 ng. The quality control (QC) profile should exhibit a primary peak within the range of 400 bp to 700 bp. Additionally, the proportion of fragments within the 900 bp to 5000 bp range should constitute more than 10% of the total. Ultimately, individual libraries were diluted to 4 nM, and then sequencing was performed on an Illumina NovaSeq. 6000 system, utilizing a strategy of 150 bp paired-end reads.

### Initial processing of raw data

Raw reads were processed to generate gene expression Matrix using SCOPE-tools v1.4.0 (Singleron). SCOPE-tools contains several sub-commands that enable the output of gene expression matrix and perform individual quality control tasks. Barcodes and UMIs were extracted from R1 reads and corrected. Adapter sequences and poly A tails were trimmed from R2 reads and the trimmed R2 reads were employed STAR v2.6.1b for the alignment of reads against the GRCm38 mouse reference genome. Initially, the reads were localized to the genome, further localized to the genes by utilizing the featureCounts software. Reads were grouped according to barcodes, UMIs, and genes, and then UMI aligned to the same gene within the same barcode were corrected, followed by UMI counting. This process yielded comprehensive Gene Expression Data, which were then compiled to form expression matrix files.

Prior to the construction of the expression matrix, a quality control filtering process was implemented for the cells. Initially, cells were selected based on a gene count criterion, retaining only those with gene counts ranging from 200 to 5,000, and cells with UMI (Unique Molecular Identifier) counts below 30,000 were excluded. Next, cells exhibiting mitochondrial RNA (mtRNA) content over 20% were removed.

### Dimension-reduction and clustering

Utilizing Seurat v3.1.2, the NormalizeData and ScaleData functions were employed to normalize and scale the gene expression levels across all cells^9^. Principal component analysis (PCA) was performed by identifying the top 2,000 variable genes using the FindVariableFeatures function. The first 20 principal components were then used to cluster cells into distinct groups using the FindClusters function. Batch effects between samples were addressed using the Harmony algorithm^10^. Finally, for the two-dimensional visualization of cellular clusters, both the Unified Manifold Approximation and Projection (UMAP) and t-distributed Stochastic Neighbor Embedding (t-SNE) were applied.

### Differentially expressed genes analysis

Differentially expressed genes (DEGs) were identified using FindMarkers function of Seurat, employing the Wilcoxon likelihood-ratio test with default parameters. DEGs in each cluster were selected based on a statistical threshold of |log2(foldchange)| ≥ 0.6 and adjusted p-values < 0.05. For comparing the CD and HFD groups, DEGs were chosen with an average |log2(foldchange)| ≥ 0.25 and adjusted p-values < 0.05.

Cell type annotations for each cluster were determined by integrating canonical marker expression, identified among the DEGs and supported by literature, and visualized using DoHeatmap, DotPlot, and VlnPlot functions of Seurat.

### Statistical analysis

Data were expressed as the mean ± standard error of mean (SEM). GraphPad Prism 8.0 (https://www.graphpad.com/scientific-software/prism/) was used to perform the statistical analysis. Student’s t test was used to compare two groups. Graphical Abstract created with BioRender.com. Data were considered statistically significant when p < 0.05.

## Data Records

All raw omics data generated in this study were deposited in the public available data base. All single cell RNA-sequencing data were deposited to the National Center for Biotechnology Information Gene Expression Omnibus database. The accession number for the single cell RNA-sequencing data of testis samples obtained from mice is GSE239391^11^. The accession numbers for the single cell RNA-sequencing data of testes from mice with a chow diet are GSM7664011, GSM7664012 and GSM7664013. The accession numbers for the single cell RNA-sequencing data of testes from mice with a HFD are GSM7664014, GSM7664015 and GSM7664016. And the files in rds format were uploaded for public (10.6084/m9.figshare.25658847.v1)^12^.

## Technical Validation

**Fig. 1:**
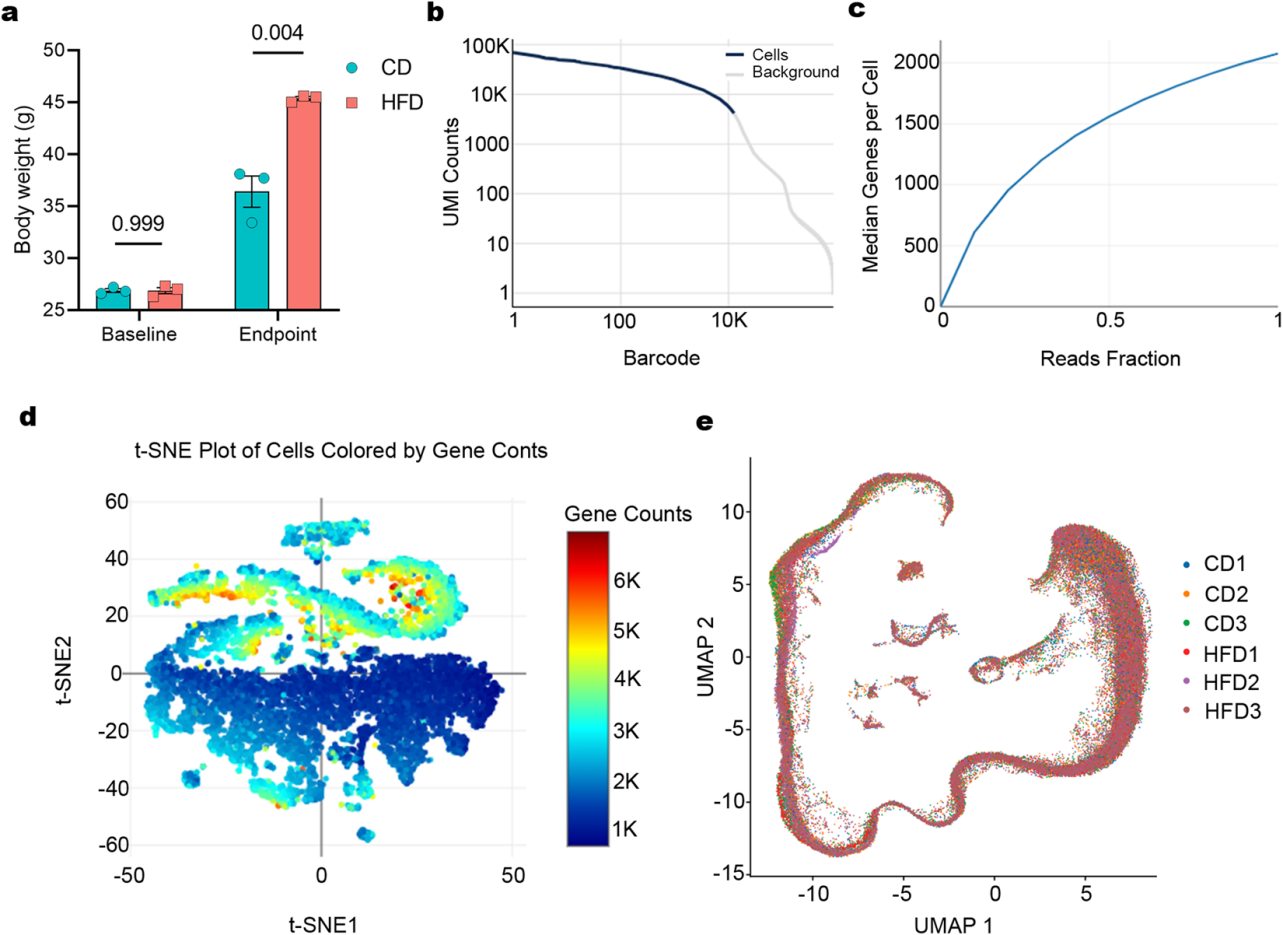
Single-cell transcriptomics of adult mouse testes with HFD and CD. (**a**) Body weight between the two groups (CD: n = 3; HFD: n = 3). (**b**) Visualization of the correlation between UMI counts and barcode detected per cell in CD1. (**c**) This curve visualizes the correlation between reads fraction and the median genes detected per cell in CD1. (**d**) t-SNE projection of cells colored by gene counts in CD1. (**e)** UMAP of testicular cell clusters.

Three testes from both CD and HFD group perform scRNA-seq (Fig. 1a). In the sequencing analysis, the saturation curve comparison revealed comparable sequencing depths across both sample sets (Fig. 1b,c, and Supplemental Figures S1-S2). Table 1 presents a comprehensive quality assessment of the sequencing data. Both groups displayed a similar total read count per sample, exceeding 110 G in data volume. The efficiency of barcodes ranged between 93.80% and 94.43%, while the Q30 Base percentage in UMI varied from 93.10% to 94.82%. Additionally, the uniquely mapped read percentages spanned from 88.84% to 90.41%. And Table 2 demonstrates consistently low mtRNA levels in cells across all samples, indicative of high cellular quality (Supplemental Figures S3). These consistent quality control metrics across various indicators suggested minimal error introduction due to operational or technical factors. Based on this, cells were filtered through a multi-step process, focusing on gene count, UMI, and mtRNA content parameters. This filtering process is essential to remove cells that do not meet the experimental criteria, thus ensuring the high quality and integrity of the dataset.

**Table 1:** Detailed quality control of FASTQ files.

Filename	Number of Reads (K)	Valid Barcodes (%)	Q30 Bases in UMI (%)	Uniquely Mapped Reads Number (%)	Base Pairs Mapped to Exonic Regions (%)
CD1	408,839	94.42	94.82	89.68	90.94
CD2	458,391	94.43	94.72	89.46	90.8
CD3	476,949	94.31	94.67	90.06	91.19
HFD1	393,219	94.07	93.43	90.41	90.4
HFD2	435,058	93.80	93.10	88.84	90.07
HFD3	433,454	94.25	94.54	89.53	91.38

**Table 2:** Statistics of Cellular mtRNA Content.

	mtRNA percent (>5%)	mtRNA percent (>10%)	mtRNA percent (>15%)	mtRNA percent (>20%)
CD1	2.11600	0.57630	0.07894	0.00000
CD2	1.86100	0.55110	0.05726	0.00000
CD3	1.62700	0.66580	0.12370	0.02854
HFD1	0.74120	0.17830	0.02815	0.00000
HFD2	1.02500	0.35580	0.03139	0.00000
HFD3	1.75200	0.29810	0.0683	0.02484

A total of 71,240 cells were examined, including 36,007 cells from the CD group and 35,233 cells from the HFD (Table 3). The fraction reads in cells was recorded between 62.83% and 68.40%. The detected gene count per cell ranged from 31,080 to 32,362, while the median gene count per cell varied from 1,980 to 2,577. Sequencing saturation levels were observed between 25.81% and 29.9%. This experiment demonstrated a high capture rate of cells per sample and an elevated median gene count per cell. Utilizing UMAP for dimensionality reduction, we projected the samples onto a two-dimensional plane, where we noted a remarkable consistency in the cellular distribution between the two groups (Fig. 1d,e). The above results indicated the robustness of sequencing data. In summary, this dataset could offer powerful tools and clear direction for advanced studies of obesity-induced modifications in the testicular microenvironment.

**Table 3:** Sequencing statistics based on cells.

Filename	Estimated Number of Cells	Fraction Reads in Cells (%)	Mean Reads per Cell	Median UMI Counts per Cell	Total Genes Detected per cell	Median Genes per Cell	Sequencing Saturation (%)
CD1	12,668	66.93	30,472	8,530	31,800	2,076	34.46
CD2	13,972	68.29	30,979	9,118	32,362	2,338	32.67
CD3	10,513	64.64	42,787	11,252	31,303	2,479	35.81
HFD1	10,658	68.40	34,707	9,982	31,080	2,160	33.88
HFD2	9,557	62.83	42,702	10,607	31,948	2,577	33.89
HFD3	16,100	62.85	25,375	6,912	31,736	1,980	29.90

**Fig. 2:**
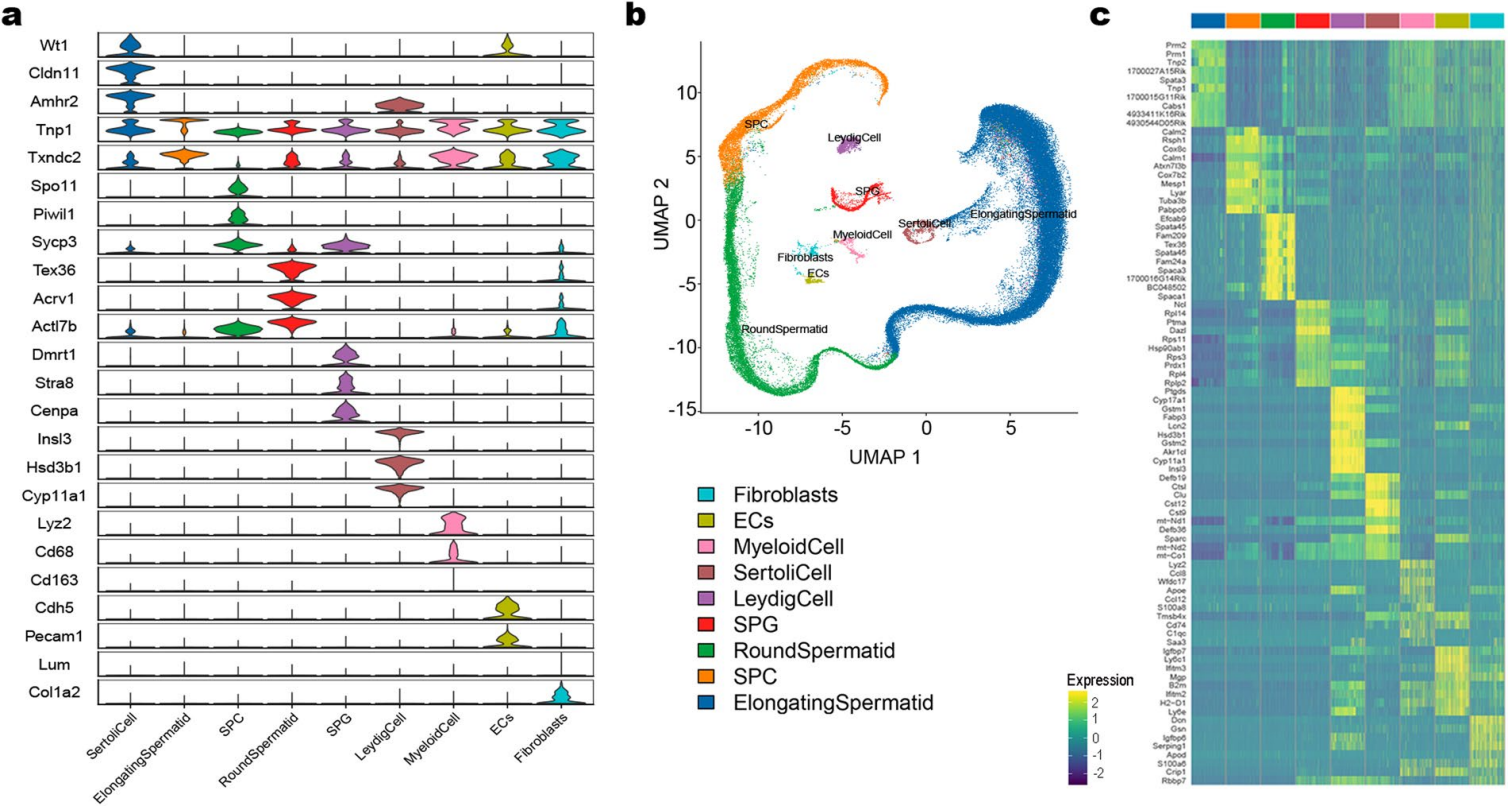
A summary of markers and characteristics of testicular cell types as delineated by scRNA-seq. (**a**) Cell-specific markers are shown by violin plot. SPG: *Dmrt1, Stra8, Cenpa*. SPC: *Spo11, Piwil1, Sycp3*. RS: *Tex36, Acrv1, Actl7b*. ES: *Tnp1, Txndc2*. SCs: *Wt1, Cldn11, Amhr2*. LCs: *Insl3, Hsd3b1, Cyp11a1*. ECs*: Cdh5, Pecam1*. Fibroblasts: *Lum, Col1a2*. Myeloid cells: *Lyz2, Cd68, Cd163*. (**b**) UMAP of single-cell transcriptomics data from testes of adult mice. Cells were classified into nine types with different colors, with each color representing one cell type. (**c**) Heatmap of the top 20 highly expressed genes in each cluster.

**Fig. 3:**
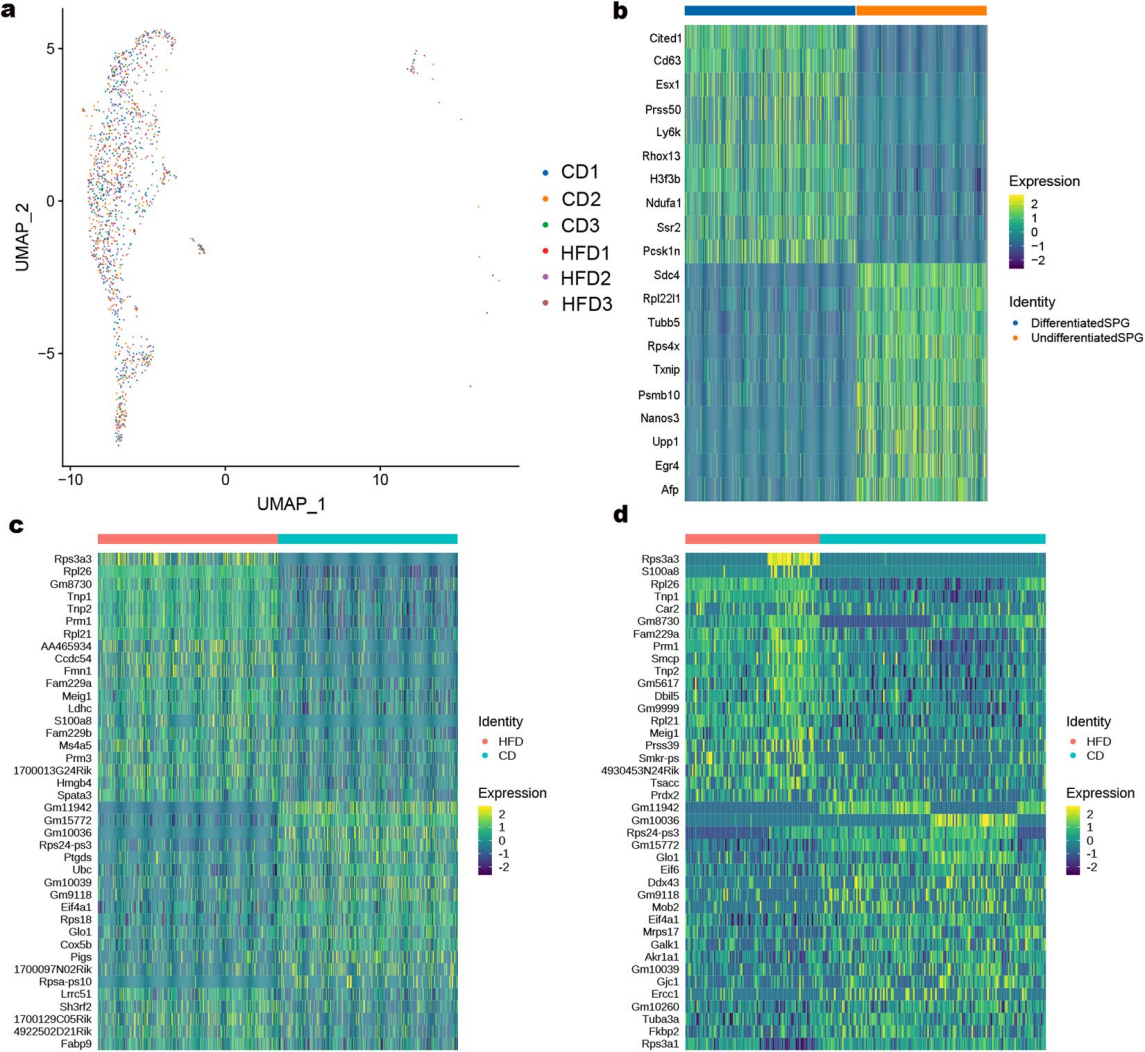
The subclusters of SPG. (**a**) UMAP of SPG in CD and HFD groups. (**b**) Heatmap of DEGs between differentiated SPG and undifferentiated SPG. Heatmap of DEGs between CD and HFD groups in differentiated SPG (**c**) and undifferentiated SPG (**d**).

**Fig. 4:**
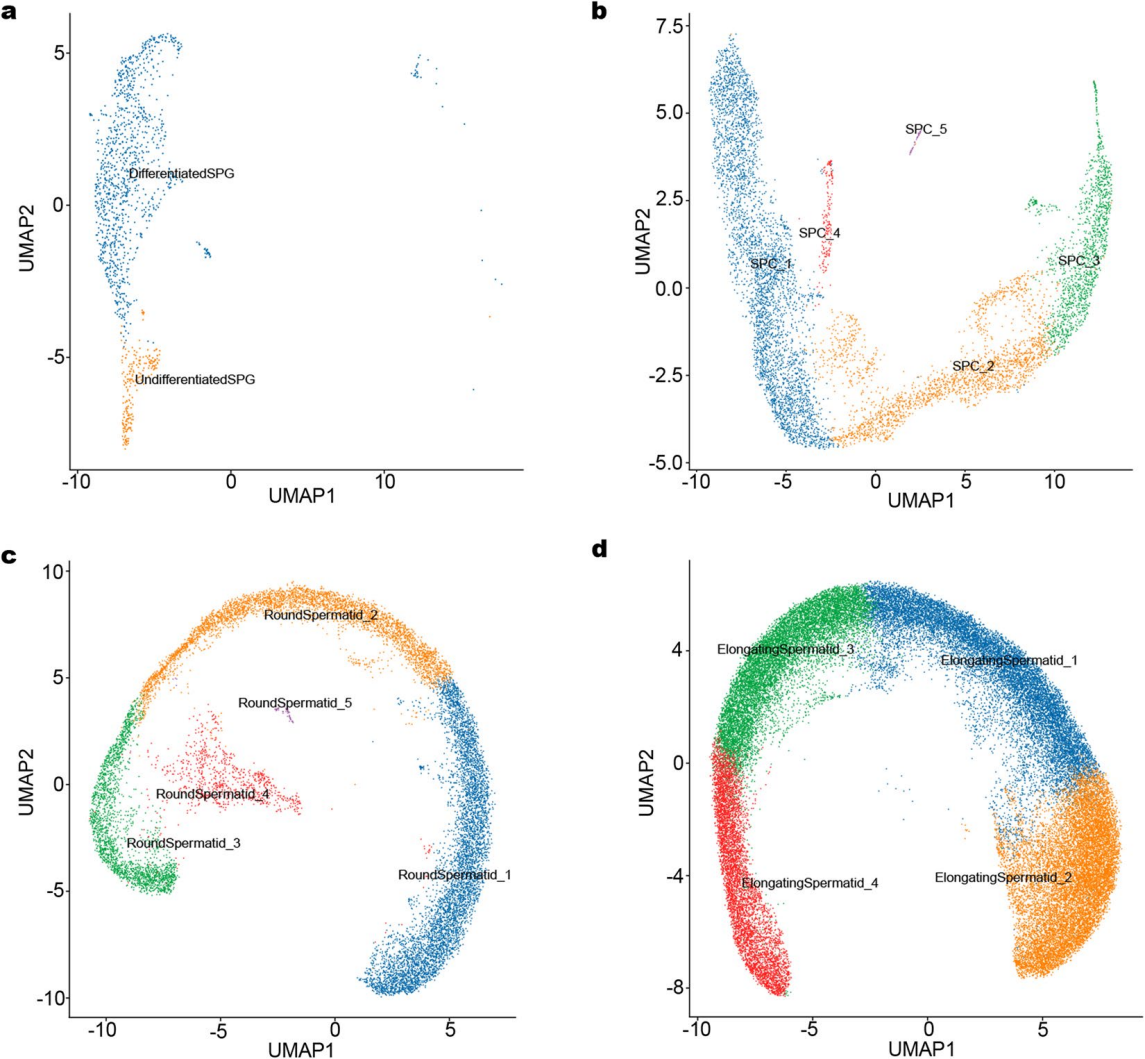
The UMAP of subclusters in SPG (**a**), SPC (**b**), RS (**c**), and ES (**d**).

**Fig. 5:**
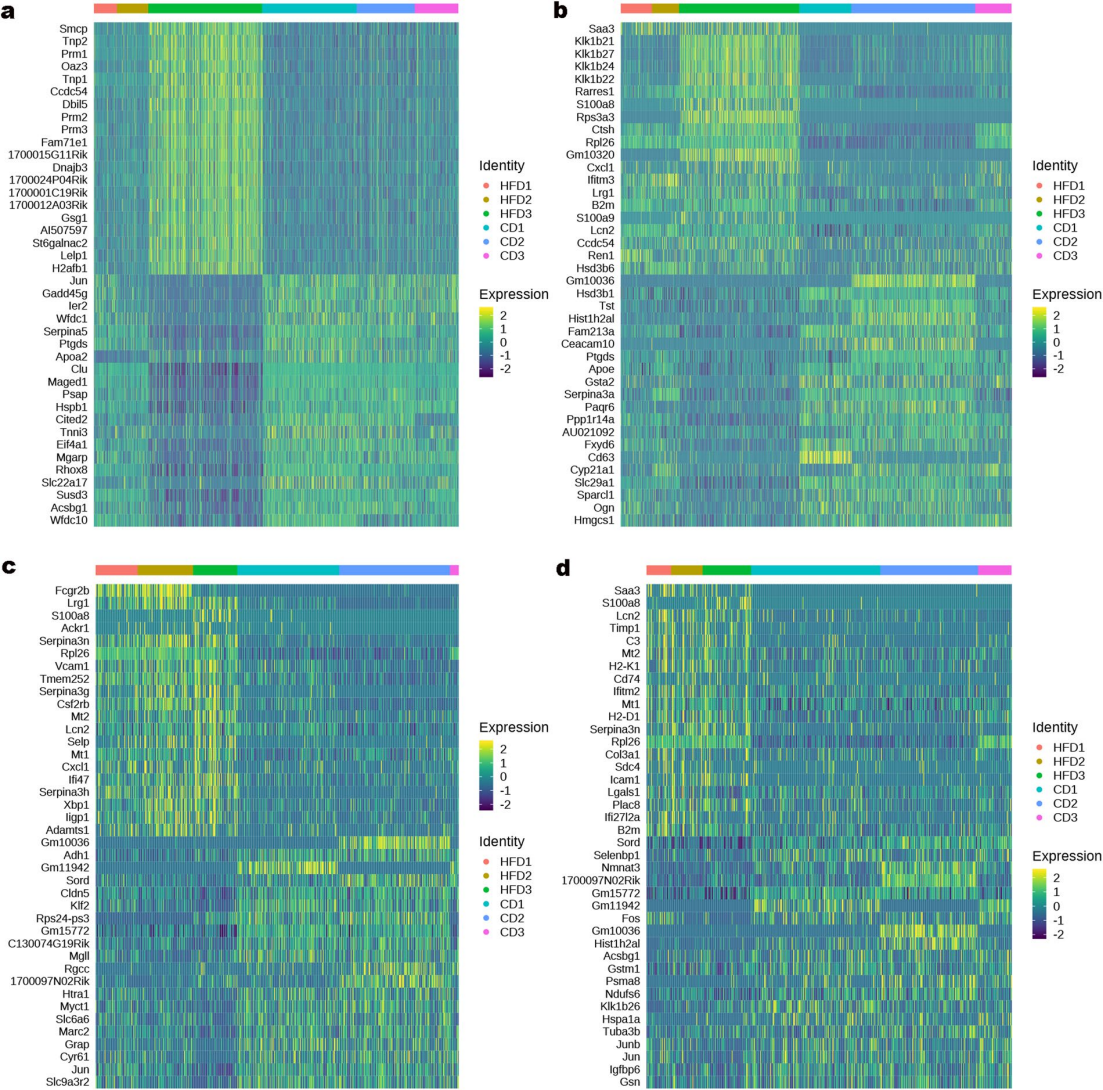
Heatmap of the top 20 highly expressed genes in each example. (**a**) SCs. (**b**) LCs. (**c**) ECs. (**d**) fibroblasts.

Based on the cell markers for mouse and human identified in previous studies (Table 4, Fig. 2a), data from application of UMAP to identify cell clusters, revealed nine major cell types: Sertoli cells (SCs), Leydig cells (LCs), endothelial cells (ECs), fibroblasts, myeloid cells, spermatogonia (SPG), spermatocyte (SPC), round spermatid (RS), and elongated spermatid (ES) (Fig. 2b). The heatmap showed the top ten DEGs in the cell cluster (Fig. 2c and Supplemental Table S1). SPG were subclustered into two distinct cell clusters (Fig. 3a and Supplemental Table S2), and transcriptional profiling revealed these groups as differentiated SPG and undifferentiated SPG, respectively marker (Fig. 3b). Analysis of genes enriched in the identified cell subclusters showed that HFD affected the transcriptome, resulting in differences between SPG in the CD and HFD groups (Fig. 3c,d, and Supplemental Table S6). Subclusters of the other spermatogenic cell depicted in Fig. 4 (and Supplemental Tables S3–S5). Furthermore, we observed that HFD altered the transcriptional profiles of testicular somatic cells (Fig. 5).

**Table 4:** Markers for each cluster were identified based on published research.

Cell type	Markers
Spermatogonia	Dmrt1, Stra8, Cenpa, Daz1
Spermatocytes	Spo11, Piwil1, Tex101, Sycp3, Spata16, Ovol2
Elongating spermatids	Prm1, Prm2, Tnp1, Spata3, Txndc2
Round spermatids	Sun5, Tex21, Tex36, Acrv1, Actl7b
Sertoli cells	Wt1, Cldn11, Amhr2
Leydig cells	Insl3, Fabp3, Hsd3b1, Cyp11a1
Endothelial cells	Cdh5, Pecam1, Vwf
Myeloid cells	Lyz2, Mrc1, Cd68, Cd163, C1qc
Fibroblasts	Lum, Dcn, Col1a2, Col1a1

In general, these preliminary processing and analyses not only demonstrate the high quality of the dataset, but also reveal the potential for exploring new spermatogenic cell subclusters, cell markers, and testicular microenvironmental changes, especially under the condition of HFD.

## Usage Notes

It is crucial to emphasize that, owing to the variance in RNA content among cell clusters in the mouse testis, normalization is a requisite step in conducting comparative analysis with this dataset. We advise implementing normalization based on either gene counts or UMI counts per cell. This guarantees data comparability, thereby substantially the precision and reliability of the results.

### Supplementary information

Supplemental Figures S1

Supplemental Figures S2

Supplemental Figures S3

Supplemental table 1

Supplemental table 2

Supplemental table 3

Supplemental table 4

Supplemental table 5

Supplemental table 6

## Data Availability

No special code was used for analysis of the current dataset. All of the analyses were done with the following open programs: SCOPE-tools (https://github.com/SingleronBio/SCOPE-tools/blob/0.1.0/scopetools/barcode.py). STAR v2.6.1b (https://github.com/alexdobin/STAR). Seurat v3.1.2 (https://github.com/satijalab/seurat).
