# Supplementary figures and images for: Single-cell transcriptome atlas of testes from mice with high-fat diets
Source: Sci Data. 2024 Jun 4;11:573. doi: 10.1038/s41597-024-03435-5 (PMC11150238; doi:10.1038/s41597-024-03435-5)

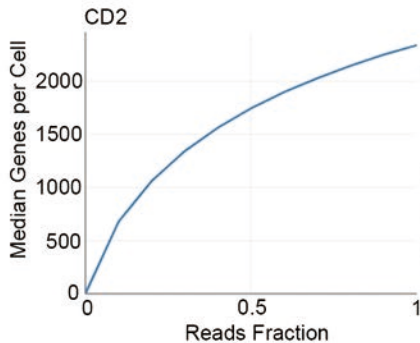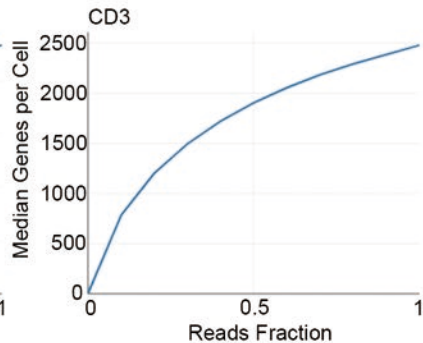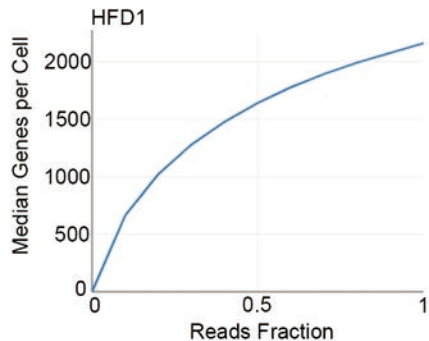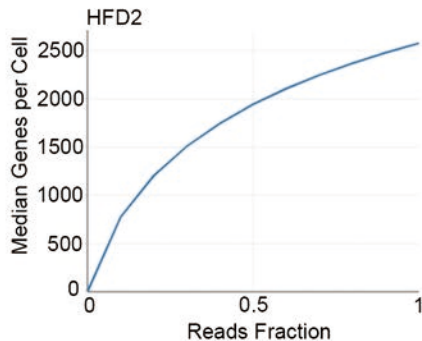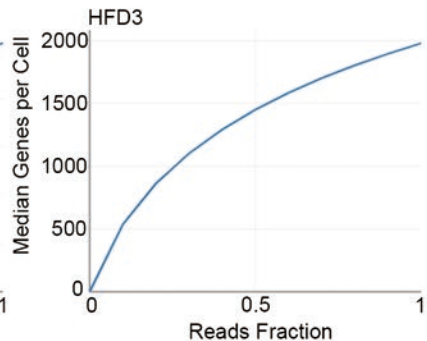

Supplement: Supplementary file 1 — Supplemental Figures S1 [file 41597_2024_3435_MOESM1_ESM.pdf]

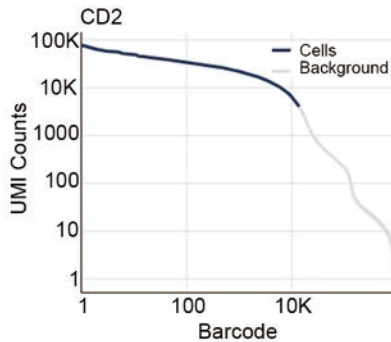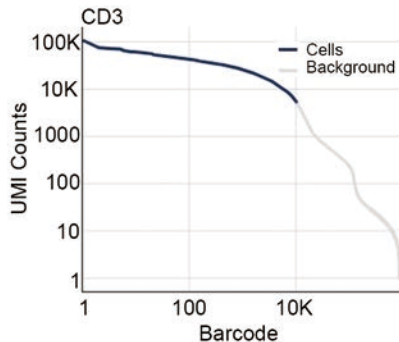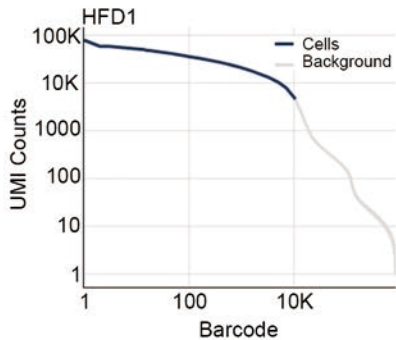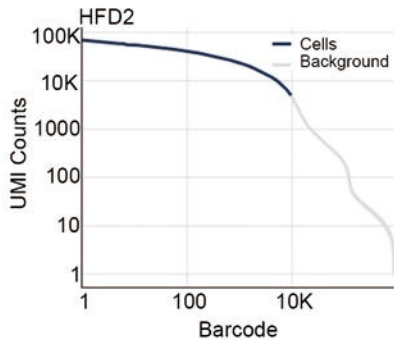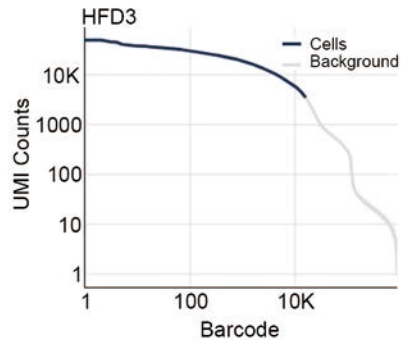

Supplement: Supplementary file 2 — Supplemental Figures S2 [file 41597_2024_3435_MOESM2_ESM.pdf]

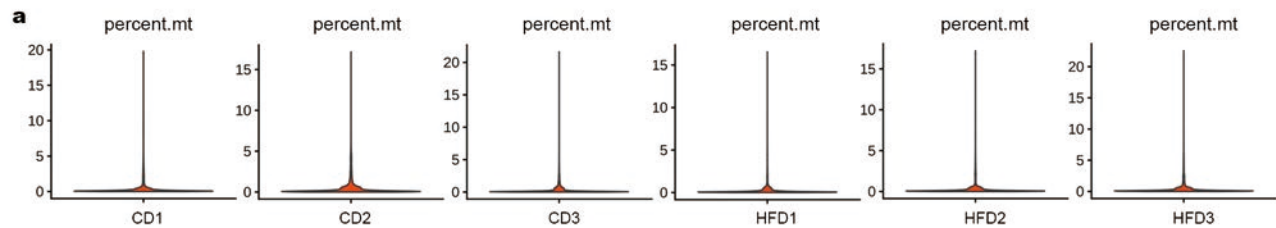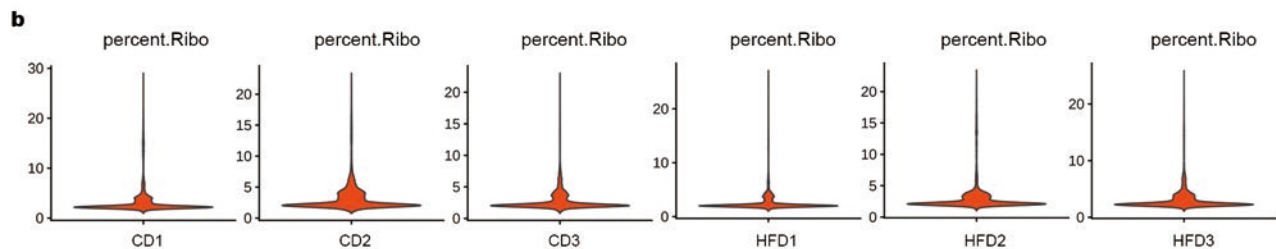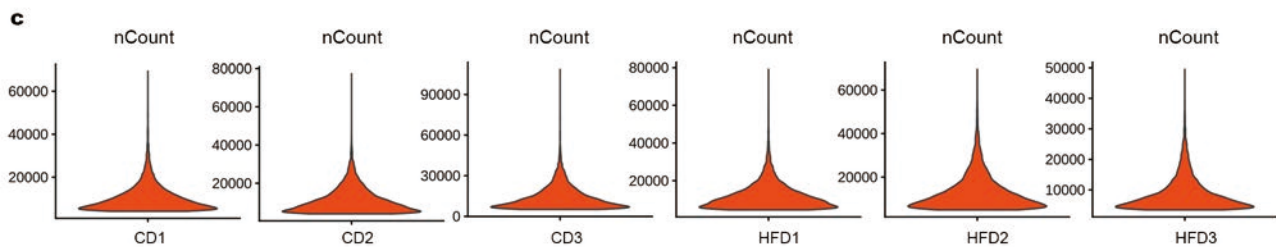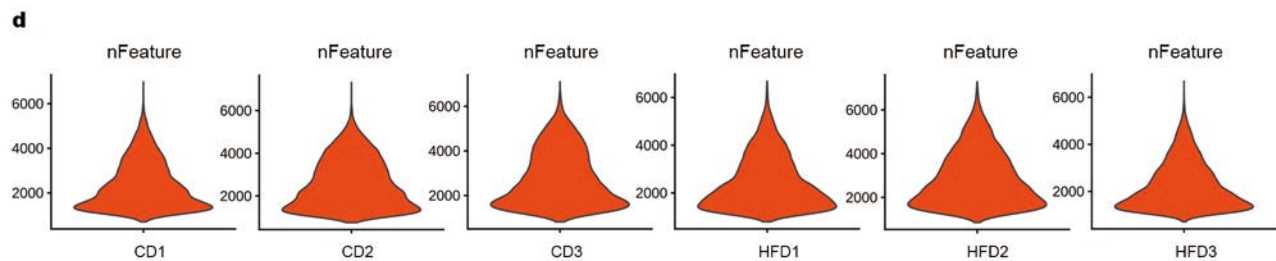

Supplement: Supplementary file 3 — Supplemental Figures S3 [file 41597_2024_3435_MOESM3_ESM.pdf]
